# Frontal and occipital brain glutathione levels are unchanged in autistic adults

**DOI:** 10.1371/journal.pone.0308792

**Published:** 2024-08-15

**Authors:** Andreia C. Pereira, Alison Leonard, Hester Velthuis, Nichol M. L. Wong, Francesca M. Ponteduro, Mihail Dimitrov, Claire L. Ellis, Lukasz Kowalewski, David J. Lythgoe, Diana-Georgina Rotaru, Richard A. E. Edden, Glynis Ivin, Charlotte M. Pretzsch, Eileen Daly, Declan G. M. Murphy, Gráinne M. McAlonan

**Affiliations:** 1 Department of Forensic and Neurodevelopmental Sciences, Institute of Psychiatry, Psychology and Neuroscience, King’s College London, London, United Kingdom; 2 Institute for Nuclear Sciences Applied to Health (ICNAS), Coimbra Institute for Biomedical Imaging and Translational Research (CIBIT), University of Coimbra, Portugal, Coimbra, Portugal; 3 Royal College of Surgeons in Ireland, Dublin, Ireland; 4 Department of Psychology, The Education University of Hong Kong, Hong Kong, China; 5 Department of Neuroimaging, Institute of Psychiatry, Psychology and Neuroscience, King’s College London, London, United Kingdom; 6 Russell H. Morgan Department of Radiology and Radiological Science, Johns Hopkins University School of Medicine, Baltimore, Maryland, United States of America; 7 F.M. Kirby Research Center for Functional Brain Imaging, Kennedy Krieger Institute, Baltimore, Maryland, United States of America; 8 South London and Maudsley NHS Foundation Trust Pharmacy, London, United Kingdom; 9 MRC Centre for Neurodevelopmental Disorders, King’s College London, London, United Kingdom; Fondazione Policlinico Universitario Gemelli IRCCS, ITALY

## Abstract

**Background:**

The neurobiological underpinnings of Autism Spectrum Disorder (ASD) are diverse and likely multifactorial. One possible mechanism is increased oxidative stress leading to altered neurodevelopment and brain function. However, this hypothesis has mostly been tested in post-mortem studies. So far, available *in vivo* studies in autistic individuals have reported no differences in glutathione (GSH) levels in frontal, occipital, and subcortical regions. However, these studies were limited by the technically challenging quantification of GSH, the main brain antioxidant molecule. This study aimed to overcome previous studies’ limitations by using a GSH-tailored spectroscopy sequence and optimised quantification methodology to provide clarity on GSH levels in autistic adults.

**Methods:**

We used spectral editing proton-magnetic resonance spectroscopy (^1^H-MRS) combined with linear combination model fitting to quantify GSH in the dorsomedial prefrontal cortex (DMPFC) and medial occipital cortex (mOCC) of autistic and non-autistic adults (male and female). We compared GSH levels between groups. We also examined correlations between GSH and current autism symptoms, measured using the Autism Quotient (AQ).

**Results:**

Data were available from 31 adult autistic participants (24 males, 7 females) and 40 non-autistic participants (21 males, 16 females); the largest sample to date. The GSH levels did not differ between groups in either region. No correlations with AQ were observed.

**Conclusion:**

GSH levels as measured using ^1^H-MRS are unaltered in the DMPFC and mOCC regions of autistic adults, suggesting that oxidative stress in these cortical regions is not a marked neurobiological signature of ASD.

## Introduction

Autism Spectrum Disorder (ASD) is an early-onset and life-long neurodevelopmental condition that affects how an individual interacts, communicates, and experiences the surrounding environment. In addition to core features, up to 70% of individuals with ASD have co-occurring neuropsychiatric and neurodevelopmental conditions, such as depression, attention deficit hyperactivity disorder (ADHD), and obsessive-compulsive disorder (OCD), resulting in a complex and heterogenous phenotype [1].

The underlying neurobiology of ASD is also complex and unclear, arising from diverse and multifactorial mechanisms. One potential mechanism is a redox system imbalance, leading to oxidative stress [2]. Markers of oxidative stress have been detected in the blood, urine, and brain of autistic individuals when compared to non-autistic (non-ASD) individuals [3]. However, it is not clear how well peripheral indices reflect differences in the brain.

The brain is susceptible to damage caused by oxidative stress, given its high energy demands and oxygen consumption (high oxidative metabolism) which produce large amounts of reactive oxygen species (ROS) [4]. It relies on neutralization by antioxidant scavenger molecules, such as glutathione (GSH) [5], the brain’s main antioxidant molecule. GSH is found in neurons and glia, with tissue levels ranging between 2–3 mM, depending on tissue type and brain region [6]. GSH captures ROS and converts to its oxidised form of glutathione disulphide (GSSG), a reaction catabolised by glutathione peroxidases. This compound is then reduced back to GSH by glutathione reductase, becoming available to the cell again and completing the redox cycle [7]. It is the constant flux between the two GSH species that allows for the continuous clearing of ROS. Reduced levels of GSH may interfere with this process and may lead to altered neurodevelopment and brain function in autistic individuals.

Evidence of widespread increased oxidative stress in the brain of autistic individuals mostly comes from post-mortem tissue [3]. Higher levels of protein oxidation markers and reduced activity of important redox enzymes responsible for GSH metabolism have been reported [3] but are not present in all brain regions [8,9]. For instance, reduced GSH and GSH/GSSG have been found in the cerebellum and temporal cortex in ASD compared to non-ASD samples [8,9], but not in frontal, parietal and occipital regions [8]. Similarly, three *in vivo* studies in adults also reported no GSH differences in frontal, occipital, and subcortical regions in autistic individuals [10–12]. In fact, sample sizes between 2500 and 6500 individuals per group would be necessary to detect a between group differences with 80% power at p<0.05 for frontal and occipital regions, respectively [12]. Thus, if a difference is present in these regions, it would be rather trivial. However, spectroscopy protocols available at the time of those studies were not tailored for GSH. Specifically, in the first two studies [10,11] the ^1^H-MRS sequences used were not designed to quantify GSH [13]. Due to its low concentration and because the GSH peak overlaps with other higher concentration molecules, the GSH signal needs to be resolved using optimized spectral editing methods [13–16]. In the only study using edited ^1^H-MRS to quantify GSH, the authors acknowledged that their GSH analysis was an exploratory component of a wider study and the quantification methodology (peak based fitting) used for GSH quantification was not optimised [12]. In addition, none of the previous studies fully accounted for potential biological-sex differences. This is problematic as the neuroimaging literature points to biological sex differences in autism [17–20], and there is preclinical evidence for sex differences in response to oxidative stress, with females potentially ‘protected’ [21].

Therefore, to address limitations of prior *in vivo* studies (including our own) and test the hypothesis that GSH levels in frontal and occipital regions are not different in the adult autistic brain, we used a GSH spectral editing sequence [14] and linear combination model fitting to improve the quantification of the captured GSH signal [22]. We quantified GSH in two regions-of-interest that overlap with previous ^1^H-MRS studies: the ‘higher-order’ cortex–dorsomedial prefrontal cortex (DMPFC), and the ‘lower-order’ cortex–the medial occipital cortex (mOCC). The DMPFC is involved in high level cognitive processes, such as social cognition, executive function, and cognitive flexibility [23–25] all of which have been implicated in autism [26]. The mOCC processes primary visual information and is crucial for the perception and integration of a range of visual features such as visuospatial integration, contrast detection and shapes recognition [27], and sensory features which are altered in autism [28–30]. We included males and females and, in addition to investigating case-control differences, we also planned correlational analyses to determine if GSH levels are related to autistic characteristics as captured by the Autism Quotient (AQ) in both groups [31]. We used the AQ to assess the extent of autistic characteristics across the autistic and non-autistic control groups in case any relationship between autism and GSH was not a simple binary (autism/no autism) difference but rather dimensional (across traits of autism); that is, to assess whether GSH levels were lower in those with more autistic characteristics on the AQ.

## Methods

This study was conducted as part of a wider series of experiments that also examined a drug response using other metrics and was approved by King’s College Research Ethics Committee (RESCM-16/17-4081) and Health Research Authority (18/WM/0208). The data presented here were acquired during a placebo (baseline) visit, when no active drug was administered. All procedures followed the Declaration of Helsinki. Participants gave written informed consent prior to any experimental procedure.

### Participants

Participants were recruited between 1 February 2018 and 20 February 2020. The general inclusion criteria for this study were age being older than 18 years and having capacity to give informed consent. Further inclusion criteria for the ASD group were an expert ICD-10 clinical diagnosis of ASD. We required a formal clinical diagnosis to be in place from a recognised ASD multidisciplinary clinical assessment setting in the UK. This included our own National Autism and ADHD service for Adults (NAASA) at the South London and Maudsley National Health Service (NHS) Foundation Trust which incorporates information from the Autism Diagnostic Interview-Revised (ADI-R) [32] where an appropriate informant is available and/or the Autism Diagnostic Observation Schedule (ADOS) [33] to inform the current symptom level. Those individuals recruited outside of our National Autism and ADHD Service for Adults (NAASA) at the South London and Maudsley NHS Foundation Trust were still carefully screened by an experienced NAASA clinician for inclusion. The clinician had to be satisfied by the account of their diagnostic assessment through a recognized UK autism service (with documentary evidence where possible). Additionally, autism traits were assessed both in non-autistic and autistic individuals using the AQ [31]. Because psychiatric medication use is frequent among autistic individuals, those under a stable dose regimen over the preceding month were also enrolled in the study. Nine autistic participants were on stable psychiatric medication regimen, antidepressants (7), antidepressants and stimulant (1), stimulant and non-benzodiazepine sleeping pills (1). General exclusion criteria were Full-Scale Intelligence Quotient (FSIQ) below 70 as measured by the Wechsler Abbreviated Scale of Intelligence (WASI-II) second edition [34]; psychiatric conditions such as: psychotic illness, major mood disorder; significant physical illness (heart disease, high blood pressure, renal insufficiency, seizures); pregnancy (all female participants took a pregnancy test at the beginning of each visit) and lactation; being on psychotropic medication (except regular medication in the ASD group as described above in the inclusion criteria for the ASD group); contraindications to magnetic resonance imaging. Over-the-counter medication was allowed (e.g., paracetamol, antihistamines). Further exclusion criteria for the ASD group were ASD caused by a known genetic syndrome, e.g., Tuberous Sclerosis, Fragile X or 22q11 deletion syndrome; those treated for epilepsy. Genetic syndromes were screened by checking patients’ records when available and during the screening interview by asking the participants themselves (and/or their carers) whether they had ever been tested or told they had a genetic condition or were aware of any testing or genetic condition within their family.

### ^1^H-MRS protocol

#### Scanning session and data acquisition

Participants were scanned on a GE Discovery MR750 3T scanner (Chicago, IL, USA) using a transmit body coil and a 12-channel head, neck and spine receive only coil at the Centre for Neuroimaging Sciences, King’s College London. A T_1_-weighted high-resolution anatomical scan was acquired for each participant. This was a sagittal Inversion-Recovery Fast Spoiled Gradient Recalled echo (IR-FSPGR) with repetition time (TR) = 7.312 ms, echo time (TE) = 3.016 ms, inversion time (TI) = 400 ms, flip angle = 11°, field of view 270 mm, 256 x 256 matrix, and 196 slices. The voxel dimensions (X, Y, Z) were: 1.055 x 1.055 x 1.2 mm. This T_1_-weighted anatomical scan was used to prescribe the ^1^H-MRS voxels during the scanning session and for voxel segmentation to allow correction of metabolite concentrations for tissue content.

We used the Hadamard Encoding and Reconstruction of MEGA-Edited Spectroscopy (HERMES) implementation which allows the simultaneous editing of γ-aminobutyric acid (GABA) and GSH [14], and while the sequence parameters are described in full for completeness, only the GSH data were included in the current work. Briefly, HERMES parameters were: TR = 2 s, TE = 80 ms, 352 averages: (88 averages for each one of four sub-experiments), 4096 data points, bandwidth 5kHz, phase cycle length of 2, 90° excitation pulses, 137° slice-selective refocusing pulses and 180° editing pulses with 20 ms duration applied at A: GABA–editing frequency = 1.9 ppm, B: GSH–editing frequency = 4.56 ppm, C: GABA+GSH–dual band editing pulse with frequencies of 1.9 ppm & 4.56 ppm and D: editing off with frequency = 7.22 ppm. A single frequency 20 ms sinc-Gaussian editing pulse was used for sub-experiments A, B and D, whereas for the simultaneous targeting of both GABA and GSH (sub-experiment C) a dual-band 20 ms cosine modulated sinc-Gaussian editing pulse was used (S1 Fig). Chemical shift selective water suppression (CHESS) was used. Auto pre-scan was run twice to ensure that water line width was <9 Hz before starting the scan. Sixteen unsuppressed water scans with the same parameters were also acquired for further water-scaling metabolite quantification and eddy current correction. Total scan duration was approximately 13 minutes for each voxel.

**Fig 1:**
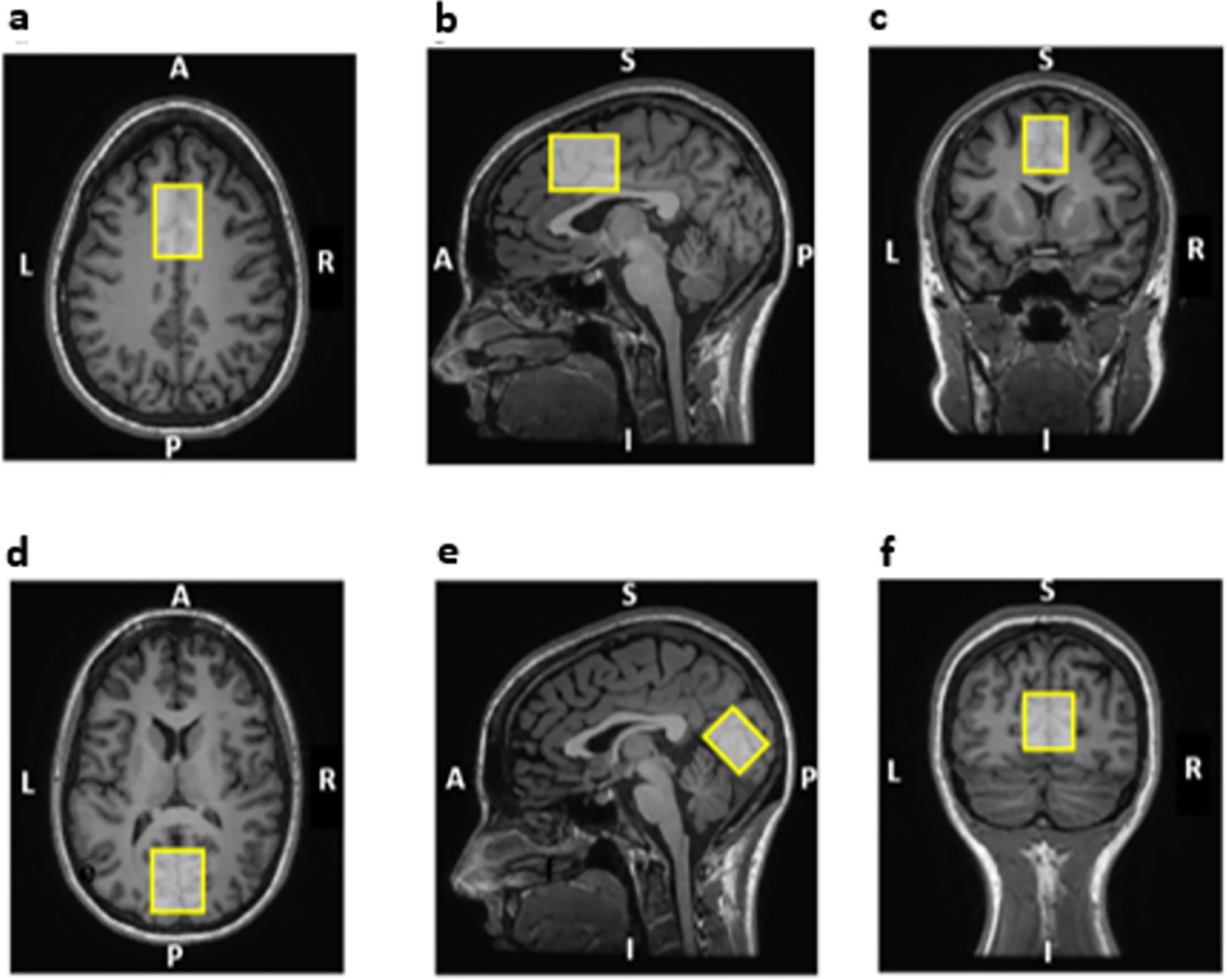
Depiction of the two 1H-MRS voxels position superimposed in the anatomical scan. Superior panel–dorsomedial prefrontal cortex voxel: a) axial view; b) sagittal view and c) coronal view. Inferior panel: Medial occipital cortex voxel–d) axial view; e) sagittal view and f) coronal view. A, anterior; P, posterior; L, left; R, right; S, superior; I, inferior.

A dorsomedial prefrontal cortex (DMPFC) voxel was prescribed aligned with the brain midline. The centre of the voxel was positioned at one third of interhemispheric fissure length (anteriorly), as measured in the first axial T_1_ slice, where no ventricles were present and avoiding the corpus callosum. The voxel mostly comprised the anterior cingulate and superior frontal cortex (dimensions rl: 25 mm; ap: 40 mm; si: 30 mm; 30 mL) (Fig 1A–1C). A medial occipital cortex (mOCC) voxel was positioned along the brain midline and parallel to the tentorium, mostly comprising the visual areas pericalcarine, cuneus, precuneus and lingual gyrus [dimensions right-left (rl): 30 mm; anterior-posterior (ap): 30 mm; superior-inferior (si): 25 mm; 22.5 mL] (Fig 1D–1F).

#### ^1^H-MRS data processing, metabolite quantification and quality assessment

Original raw P-files were exported from the scanner and run through in-house scripts adapted from FID appliance (FID-A) pre-processing pipeline [35] running in MATLAB 9.2.0 (R2017a, The Mathworks Inc., Natick, Massachusetts, USA). Preprocessing steps included weighted receiver coil combination, removal of motion corrupted averages, frequency and phase drift correction, and spectral registration to align the four sub-spectra prior to subtraction to minimise subtraction artifacts [36,37]. The GSH difference spectrum GSH_diff_ was calculated from the Hadamard-encoded sub-spectra as GSH_diff_ = B + C–A–D. The pre-processed GSH_diff_ and water unsuppressed spectra (used for eddy current correction and water-scaled metabolite quantification) were fed into LCModel version 6.3-IL (Stephen Provencher Inc.). The basis set used in the current study was simulated with FID-A software and high-spatial resolution density matrix simulations using the fast 1D projection method [38,39]. The individual metabolite spectra were obtained using published chemical shifts and J-coupling constants [40]. Radiofrequency pulse shape and timings for our HERMES implementation were used for the simulations, providing optimised spectral fitting and quantification. The simulated basis set included glutathione, n-acetylaspartate and n-acetylaspartilglutamate. The ppm window for analysis was 1.9 ppm– 3.6 ppm.

Each individual spectrum was visually inspected for artifacts (subtraction artifacts, ghosts), baseline irregularities and residuals. The threshold for the Cramer-Rao lower bounds (%CRLB) error estimate was set to < 20% as suggested by the LCModel manual. Importantly, the signal to noise ratio (SNR) and spectral line width are important indicators of data quality [41,42] and are, thus, reported. Both the quality procedures and measures reported follow published guidelines [36,42–44].

Each voxel was co-registered to the IR-FSPGR anatomical scan using the standalone co-registration routine from Gannet 3.0 (Edden et al., 2014) (http://www.gabamrs.com/downloads) running in MATLAB 9.2.0 (The Mathworks Inc., Natick, Massachusetts, USA). It uses the Statistical Parametric Mapping 12 (SPM12) [45] (https://www.fil.ion.ucl.ac.uk/spm/software/spm12/, University College London, UK) segmentation tool to extract the proportion of grey matter (pGM), white matter (pWM) and cerebrospinal fluid (pCSF) within the voxel which were then used to correct metabolite values for partial volume effects and different amounts of ‘visible’ water in each tissue type (accounting for possible confounds of different tissue proportions within the voxel) which could confound the analyses. Each individual metabolite was corrected using the following calculation:

$$Met_{corr}=Met_{LCM}\frac{(43300*pGM+35880*pWM+55556*pCSF)/35880}{1-pCSF}$$ (1)

where Met_corr_ is the corrected metabolite value, Met_LCM_ is the original metabolite value obtained from LCModel, 43300, 35880, and 55556 are the concentrations (in mM) of water in GM, WM, and CSF, respectively [46]. The division by 35880 in the numerator corrects for the initial LCModel analysis that assumes a purely white matter voxel during quantification [47]. Finally, the attenuation factor of water at TE = 80 ms was accounted for during quantification by inputting ATTH2O = 0.37 in the analysis parameters [47]. No other T_1_ or T_2_ relaxation times were considered, thus, metabolites are reported in institutional units (iu).

### Statistics

We used IBM SPSS statistics version 25 for Windows (IBM Corporation, IL, USA) for statistical analysis. GraphPad Prism 9.4.1 for Windows (GraphPad Software, San Diego, California, USA, http://www.graphpad.com) for figures creation.

We checked whether our data had a normal distribution using the Shapiro-Wilk’s test. Next, we used either parametric T test for normally distributed data or non-parametric Mann-Whitney test for non-normally distributed data for between group comparisons of demographics, clinical, behavioural, and ^1^H-MRS data. We used Fisher’s exact test to compare the proportion of males and females between the two groups. We ran a univariate ANOVA with group and sex as independent variables and GSH levels as dependent variables separately for each region to test for a main effect of sex and a group x sex interaction. Since some autistic individuals were taking medication, we also ran a separate univariate ANOVA with group and medication (i.e. “yes “or “no”) as independent variables and GSH as dependent variable to test for a main effect of medication for each region separately. Where a medication effects was found, we divided the ASD group into subgroups ASD medicated (ASD_M) and ASD non-medicated (ASD_NM). We then compared GSH level between ASD_M, ASD_NM and non-autistic participants using a univariate ANOVA. We ran correlations between GSH and AQ both across and within each group separately using either Pearson or Spearman correlations for normally and non-normally distributed variables, respectively. The significance threshold was set to α = 0.05. We extracted the effect sizes directly from SPSS. When these were not directly available we calculated them at https://www.psychometrica.de/effect_size.html [48] (for non-parametric tests).

## Results

### Demographic information

Thirty-one adult autistic participants (24 males, 7 females) and 40 non-ASD controls (21 males, 19 females) were included, making this the largest ASD sample to date. Demographic information, namely age, biological sex, FSIQ, AQ, ADOS and ADI-R scores are summarised in Table 1. Briefly, the groups did not differ in FSIQ. The proportion of males: females differed between groups, with a smaller proportion of females in the ASD group. Age differed between the two groups, such that the ASD group was on average ~ 5 years older than the non-ASD group. However, since age did not correlate with GSH levels in either region (both p > 0.339) it was not further considered in any analysis. As expected, AQ scores differed between groups, with the ASD group scoring higher (i.e. higher symptom incidence) than the non-ASD group.

**Table 1:** Participants’ demographics and neuropsychological scores.

		non-ASD		ASD		Statistics
	mean ± SD [95% CI]	Range	mean ± SD [95% CI]	Range		
N (m/f)	40(21/19)	--	31(24/7)	--	e)	**p = 0.046**
Age	30 ± 11 [27 –33]	18–58	35 ± 11 [31 – 39]	19–51	U = 789.5	**p = 0.049**
FSIQ^a^	120 ± 10 [117–123]	100–138	116 ± 9 [113–120]	100–134	*t*(40) = 1.435	p = 0.156
AQ^b^	16 ± 8 [13 – 19]	3–36	36 ± 8 [33 – 40]	21–48	*t*(58) = -9.730	**p < 0.001**
ADOS^c^						
Communication	----	----	4 ± 2 [3 – 5]	1–8	----	----
Soc Interaction	----	----	7 ± 2 [6 – 8]	3–12	----	----
ADI^d^						
Communication	----	----	12 ± 8 [6 – 18]	0–23	----	----
Soc Interaction	----	----	16 ± 10 [8 –23]	1–28	----	----
Rep Behaviours	----	----	5 ± 3 [2 –7]	0–8	----	----

Abbreviations: non-ASD, non-autistic control group; ASD, autism spectrum disorder group; n, number of participants; SD, standard deviation; 95% CI, 95% confidence interval; N, number of participants; m, male, f, female; FSIQ, full-scale intelligence quotient; AQ, Autism-Spectrum Quotient; ADOS, Autism Diagnostic Observation Scale; ADI-R, Autism Diagnostic Interview-Revised; U, Mann-Witney test value; *t*, t test value; ^a^data available from 35 non-ASD and 27 ASD participants

^b^data available from 35 non-ASD and 25 ASD participants

^c^data available from 19 ASD participants

^d^data available from 9 ASD participants

^e^ Fisher’s exact test; p, statistical value. Statistically significant group differences in bold (p < 0.05).

### ^1^H-MRS quality assessment and voxel tissue composition

Following thorough visual inspection, the included spectra had signal-to-noise ratio (SNR) ranging between 4 and 8, linewidth between 0.010 ppm (~ 1 Hz) and 0.105 ppm (~ 13 Hz), and % CRLB between 7 and 22. An example of a GSH difference spectrum for the DMPFC and the mOCC voxels can be found in S2 Fig. There were 31 non-ASD and 22 ASD datasets available for the DMPFC region and 38 non-ASD and 28 ASD datasets available for the mOCC region. S1 Table summarises quality indices and GSH levels in institutional units, and S2 Table summarises voxel tissue composition. Briefly, there were no between group differences in any quality indicators, except for a statistically significant difference in the DMPFC SNR (U = 289.5, p = 0.008) that was only slightly higher in the non-ASD group (mean ± SD_non-ASD_ = 6.3 ± 1 vs mean ± SD_ASD_ = 5.5 ± 1). There were no between group differences regarding voxel tissue composition.

### GSH

**Fig 2:**
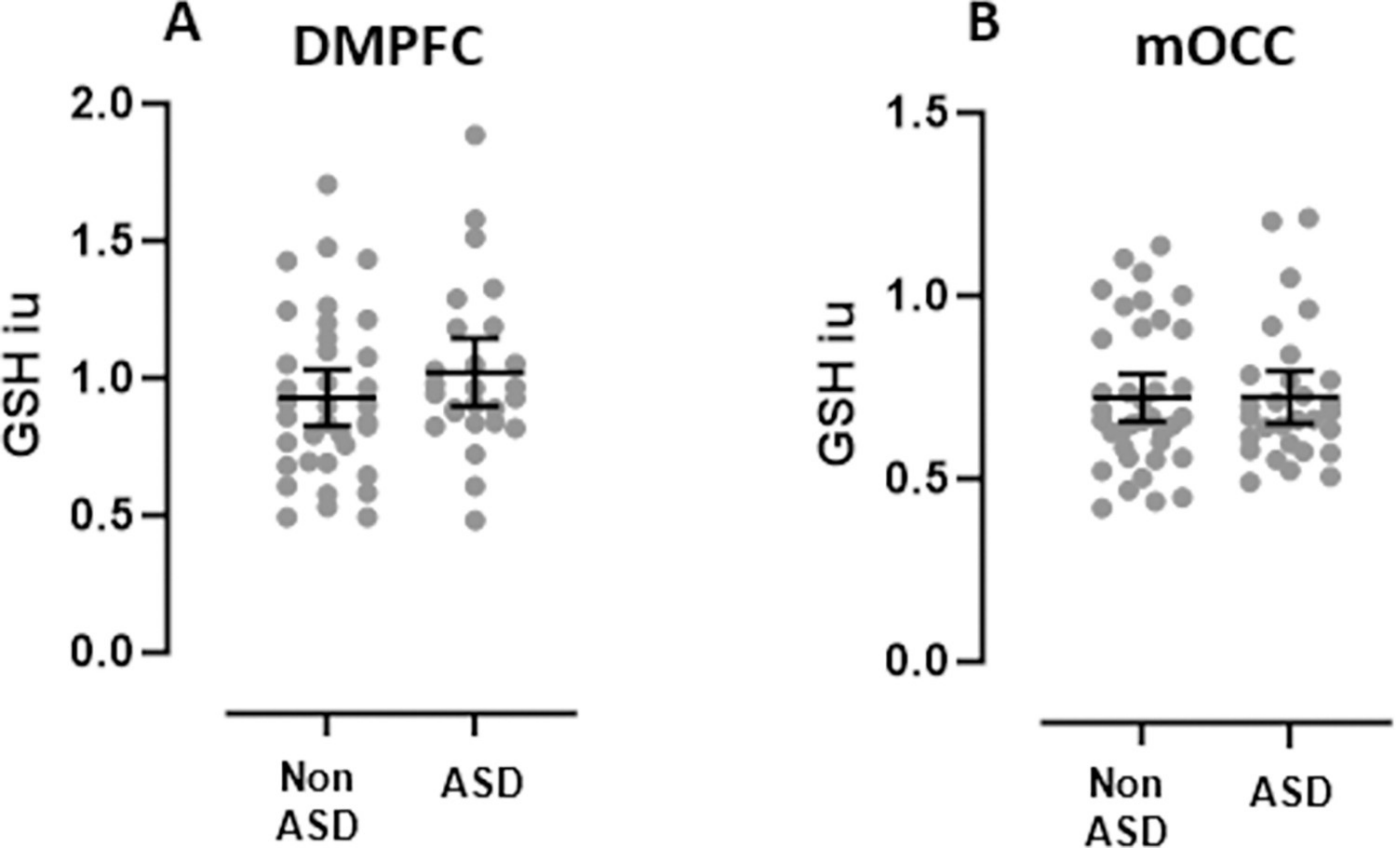
GSH levels in A) DMPFC and B) mOCC regions. There were no between group differences in GSH levels in either region. Dots represent individual metabolite values and lines represent mean ± 95% confidence interval. Abbreviations: DMPFC, dorsomedial prefrontal cortex; mOCC, medial occipital cortex, GSH, glutathione; iu, institutional units; non-ASD, non-autistic group; ASD, Autism Spectrum Disorder group.

There were no between group differences in GSH levels either in the DMPFC (ASD: 1.022 ± 0.304 vs non-ASD: 0.928 ± 0.300, U = 559, p = 0.194, η^2^ = 0.027, observed power = 0.2) or mOCC (ASD: 0.722 ± 0.188 vs non-ASD: 0.722 ± 0.198, U = 558, p = 0.929, η^2^ < 0.001, observed power = 0.05) regions (Fig 2).

Regarding the exploratory sex effect analyses, there were no significant main effects of group (F_(1, 58)_ = 2.877, p = 0.095, η^2^ = 0.047), sex (F_(1, 58)_ = 2.623, p = 0.111, η^2^ = 0.043) or group x sex interaction (F_(1, 58)_ = 0.557, p = 0.459, η^2^ = 0.010) in the DMPFC region. Similarly, in the mOCC there were no main effects of group (F_(1, 63)_ = 0.005, p = 0.946, η^2^ < 0.001), sex (F_(1, 63)_ = 0.015, p = 0.901, η^2^< 0.001) or group x sex interaction (F_(1, 63)_ = 0.013, p = 0.910, η^2^ < 0.001).

Finally, when considering medication, there was no significant effect of medication in the mOCC (F_(1, 64)_ = 1.241, p = 0.269, η^2^ = 0.019). However, there was a significant effect of medication in the DMPFC (F_(1, 59)_ = 5.116, p = 0.027, η^2^ = 0.08). A follow-up ANOVA comparing the DMPFC GSH level of the subgroups ASD medicated (ASD_M), ASD non-medicated (ASD_NM) and non-ASD confirmed that there were significant differences in mean GSH between the groups (F_(2,59)_ = 3,326, p = 0.043, η^2^ = 0.101). Specifically, GSH levels of the ASD_M group were significantly higher than the non-ASD group (ASD_M: 1.22 ± 0.36 vs non-ASD: 0.93 ± 0.30, p = 0.015, η^2^ = 0.097), as well as than ASD_NM (ASD_M: 1.22 ± 0.36 vs ASD_NM: 0.94 ± 0.24, p = 0.027, η^2^ = 0.08). However, the main results did not materially change when medicated participants were excluded (non-ASD: 0.93 ± 0.30 vs ASD_NM: 0.94 ± 0.24, p = 0.936, η^2^ < 0.001, observed power = 0.05).

### Correlations with Autism Quotient scores

We observed no correlations between GSH levels and AQ in either region either across groups or in each group separately. Excluding data from individuals taking medication did not change the results.

## Discussion

We quantified GSH levels of the DMPFC and OCC regions in adults with and without ASD using ^1^H-MRS spectral editing and an optimised quantification methodology that allowed us to improve GSH detection, overcoming previous ^1^H-MRS studies’ limitations.

We acknowledge that this pulse sequence permits the measurement of many more metabolites than GSH, and specifically GABA [14]. However, the goal of the present analyses was to provide clarity on any potential alteration in GSH and hence oxidative stress in autism. This does not detract from the importance of GABA in autism and this literature has been comprehensively reviewed in recent meta-analyses which showed that GABA levels are reduced in limbic, frontal and temporal brain regions in autism [49,50].

On this measure, differences in oxidative metabolism differences were not evident. Additionally, we observed no effects of biological sex on GSH levels either across or between groups and no relationship between GSH and current autism scores as measured by AQ. We observed an effect of medication in the DMPFC region; medicated autistic participants had higher GSH than non-autistic and non-medicated autistic participants.

Our findings are in line both with previous post-mortem [8] and *in vivo* [10–12] literature regarding frontal and occipital GSH levels in ASD. Thus, taken together, GSH levels in frontal and occipital regions are not altered in autistic adults. However, our findings do not exclude the possibility that there are GSH alterations elsewhere in the autistic brain. For example, two post mortem studies reported lower GSH in temporal and cerebellar regions in ASD [8,9], therefore GSH differences in ASD may well be brain-region specific. The reasons for this may be diverse. For instance, this could reflect complex differences in regional cellular composition and antioxidant enzyme distribution [51,52]. Hence, future studies should investigate *in vivo* GSH levels in other regions, especially in the temporal and cerebellar cortex [8,9].

Alternatively, it is also possible that altered redox regulation might be present only in distinct subgroups of autistic individuals. For example, mitochondrial dysfunction has been proposed as a source of increased oxidative stress in ASD [53] but within a distinct subgroup [54], or associated with co-occurring epilepsy [55].

Age is also potentially important. While an *in vivo* study in children with ASD reported no GSH differences in frontal, motor, and thalamic regions[56], post-mortem research point to indices of altered brain energy metabolism and oxidative stress in temporal, cerebellar, and frontal brain samples of autistic children that were not detected in adult samples [57]. Both brain and peripheral (blood and urine) markers of increased oxidative stress have also been associated with higher symptom severity in autistic children, in particular those with more challenging clinical presentations [58–60]. The autistic individuals included in the current and previous ^1^H-MRS studies of GSH [10–12] were adults with average range intellectual ability and no comorbid epilepsy, which might partially explain why GSH levels were unaltered in these studies.

Despite no overall between-group mean GSH differences, there was some evidence for a medication effect in this study–autistic people taking medication had higher GSH levels in the DMPFC region than non-medicated and non-autistic individuals. We cannot say whether higher GSH levels in the (small) medicated group were explained by greater biological complexity associated with co-occurring mental health difficulties or a consequence of medication or both. However, sertraline (taken by the 6 out of 9 of the medicated ASD participants in our sample) has been reported to increase GSH levels in the brain of mice [61]. Future studies in larger samples should examine potential impact of medication on oxidative metabolism in ASD.

We did not find any evidence for a sex-effect on GSH levels in either region. Preclinical literature points to females being less susceptible to oxidative stress than males [21]. While neurochemical sex effects have been reported already for other metabolites in autistic individuals [19,20], our study suggests that these do not extend to GSH. However, we acknowledge that our exploratory analysis had relatively low numbers of female participants and larger sample sizes may be needed to detect sex-specific effects in general, and specifically in ASD [62]

We observed no correlations between GSH and AQ scores in either region. This is partially in line with a previous study from our group where GSH levels in an overlapping dorsomedial prefrontal region were not correlated with ADOS scores in adults with ASD. The AQ assesses five different behavioural areas in which the DMPFC and OCC are involved: social skills, attention switching, attention to detail, communication, and imagination, being a tool to assess where an individual falls on the spectrum between autism and typical behaviour [31]. That we did not find even correlational evidence for a link between GSH and this range of autistic traits supports the hypothesis that in adulthood, autistic presentation is not contributed to by underlying alterations in frontal and occipital GSH levels.

### Limitations

The most common physiological marker for oxidative stress is decreased GSH/GSSG ratio [5,63]. However, biological levels of GSSG are below detection limits of ^1^H-MRS [64]. Hence, we cannot rule out the possibility that GSSG levels, and consequently GSH/GSSG ratio, were altered in the current autistic cohort. While post-mortem studies of ASD reported decreased GSH/GSSG alongside decreased GSH [8,9], whether this measure alone will also be meaningful in *in vivo* studies in ASD needs to be further investigated. We included only adults with normal to high intelligence, therefore our results do not generalise to the whole spectrum of autism. As discussed, GSH alterations may be present in cohorts with different cognitive and age profiles. Lastly, as expected, the recruitment of autistic females was more challenging than males and, in this study, only seven autistic females were included, corresponding to a 3:1 male:female ratio in the ASD group. This male:female ratio is typical in ASD [65], and although previous studies often excluded females, we acknowledge that our analysis of a potential sex effect is still underpowered and should be specifically addressed in future studies.

## Conclusions

The current work confirmed that GSH levels in the DMPFC and mOCC regions are not altered in autistic adults. This suggests that in this sample, any differences in the redox machinery of the brain associated with ASD does not extend to a shift in GSH anti-oxidant levels as measured by ^1^H-MRS.

## Supporting information

S1 Fig Single-voxel HERMES pulse sequence diagram. A) depicts the single-band editing pulses used for GSH-only, GABA-only, and edit OFF; B) depicts the dual-band pulses for simultaneously editing GSH and GABA.
(DOCX)

S2 Fig Example of a glutathione (GSH) difference spectrum from the A) dorsomedial prefrontal cortex (DMPFC) and B) from the medical occipital cortex (mOCC).
(DOCX)

S1 Table ^1^H-MRS quality measures and GSH levels in institutional units for each voxel and group. (DOCX)

S2 Table Voxel tissue proportions for each region and group. (DOCX)

S1 Data Study dataset. (XLSX)

## Abbreviations

ADHD: attention deficit hyperactivity disorder
ADI-R: autism diagnostic interview-revised
ADOS: autism diagnostic observation schedule
ANOVA: analysis of variance
AQ: autism quotien
ASD: autism spectrum disorder
CHESS: chemical shift selective water suppression
CRLB: Cramer-Rao lower bounds
CSF: cerebrospinal fluid
DMPFC: dorsomedial prefrontal cortex
FSIQ: full-scale intelligence quotient
GABA: γ-aminobutyric acid
GM: grey matter; GSH, glutathione
GSSG: glutathione disulphide
HERMES: Hadamard Encoding and Reconstruction of MEGA-Edited spectroscopy
ICD-10: international classification of diseases tenth revision
^1^H-MRS: proton-magnetic resonance spectroscopy
mOCC: medial occipital cortex
OCD: obsessive-compulsive disorder; ppm, parts per million
ROS: reactive oxygen species
SD: standard deviation
SNR: signal to noise ratio
IR- FSPGR: inversion-recovery fast spoiled gradient recalled echo
SPSS: statistical package for social sciences
TE: echo time
TI: inversion time
TR: repetition time
WASI-II: Wechsler Abbreviated Scale of Intelligence second edition
WM: white matter
