# Supplementary material for: Frontal and occipital brain glutathione levels are unchanged in autistic adults
Source: PLoS One. 2024 Aug 15;19(8):e0308792. doi: 10.1371/journal.pone.0308792 (PMC11326623; doi:10.1371/journal.pone.0308792)
Supplement: S1 Fig — A) depicts the single-band editing pulses used for GSH-only, GABA-only, and edit OFF; B) depicts the dual-band pulses for simultaneously editing GSH and GABA. (DOCX) [file pone.0308792.s001.docx]

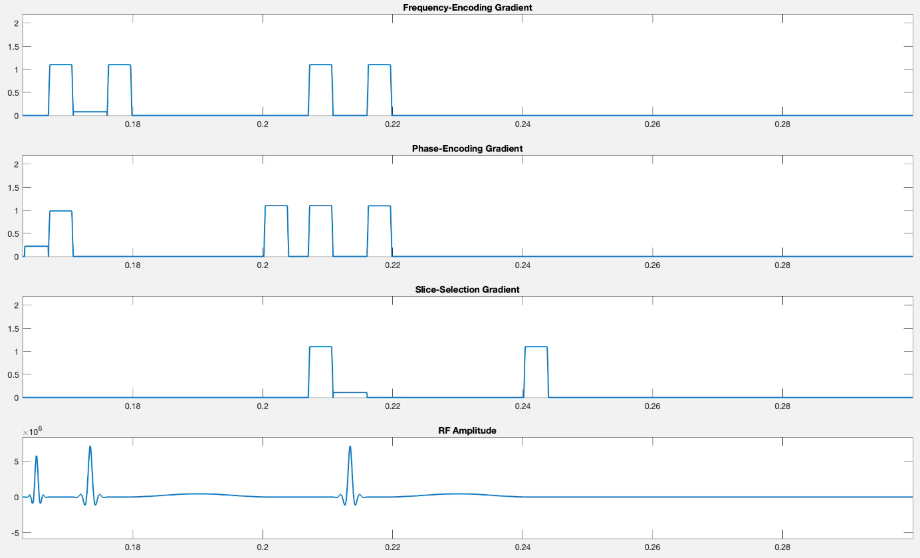

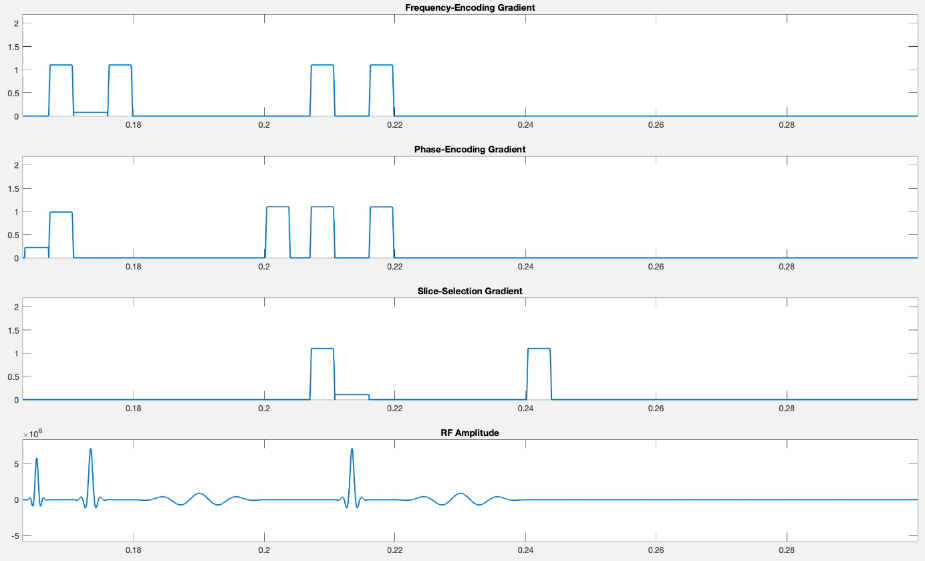


**A**

**B**

**S1 Figure. Single-voxel HERMES pulse sequence diagram**. A) depicts the single-band editing pulses used for GSH-only, GABA-only, and edit OFF; B) depicts the dual-band pulses for simultaneously editing GSH and GABA.
