# Supplementary material for: Frontal and occipital brain glutathione levels are unchanged in autistic adults
Source: PLoS One. 2024 Aug 15;19(8):e0308792. doi: 10.1371/journal.pone.0308792 (PMC11326623; doi:10.1371/journal.pone.0308792)
Supplement: S2 Fig — Example of a glutathione (GSH) difference spectrum from the A) dorsomedial prefrontal cortex (DMPFC) and B) from the medical occipital cortex (mOCC). (DOCX) [file pone.0308792.s002.docx]

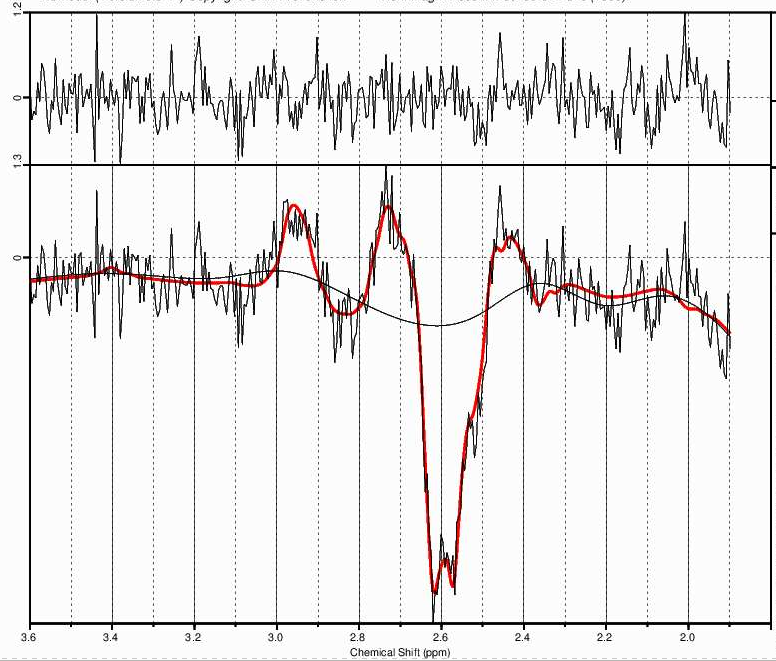

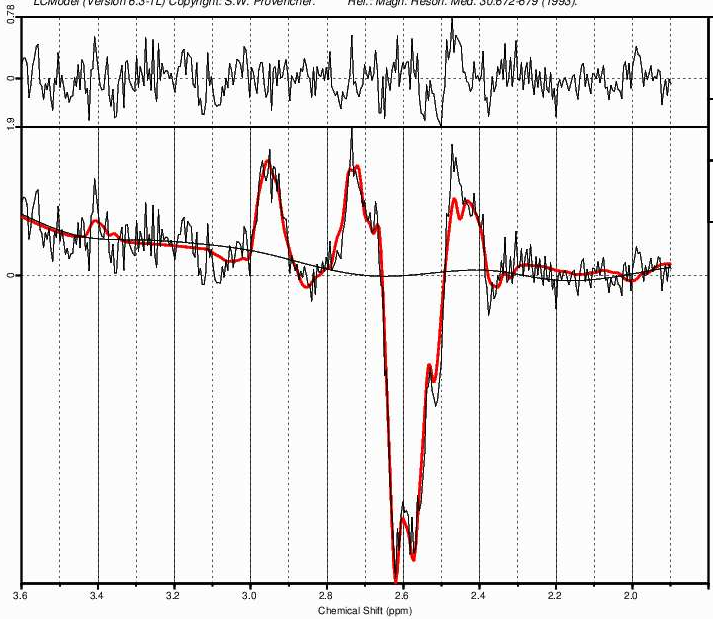


mOCC

DMPFC

**B**

**A**

GSH

GSH

**S2 Figure**. **Example of a glutathione (GSH) difference spectrum from the A) dorsomedial prefrontal cortex (DMPFC) and B) from the medical occipital cortex (mOCC).**
