# Supplementary material for: Frontal and occipital brain glutathione levels are unchanged in autistic adults
Source: PLoS One. 2024 Aug 15;19(8):e0308792. doi: 10.1371/journal.pone.0308792 (PMC11326623; doi:10.1371/journal.pone.0308792)
Supplement: S1 Table — (DOCX) [file pone.0308792.s003.docx]

**S1 Table**. **^1^H-MRS quality measures and GSH levels in institutional units for each voxel and group.**

|  |  |  | non-ASD |  | ASD |  | Statistics | | |
| --- | --- | --- | --- | --- | --- | --- | --- | --- | --- |
| Region |  |  | mean ± SD 95% CI | Range | mean ± SD 95% CI | Range | |  |  |
|  | N (m/f) |  | 36 (19/17) | -- | 26 (20/6) | -- |  | |  |
|  | GSH |  | 0.93 ± 0.3 [0.83-1.03] | 0.49 - 1.7 | 1.0 2± 0.3 [0.90-1.14] | 0.48 - 1.89 | U = 377.0 | | p = 0.097^a^ |
| DMPFC | SNR |  | 6.3 ± 1 [6 -7] | 4 - 8 | 5.5 ± 1 [5 -6] | 4 - 8 | U = 289.5 | | **p = 0.008** |
|  | FWHM |  | 0.05 ± 0.02 [0.04 – 0.05] | 0.010 - 0.105 | 0.05 ± 0.02 [0.04 – 0.05] | 0.02 – 0.09 | U = 437.5 | | p = 0.662 |
|  | % CRLB GSH |  | 10 ± 2 [10 – 11] | 8 - 16 | 11 ± 2 [10 – 12] | 7 - 18 | U = 450.0 | | p = 0.794 |
|  | N (m/f) |  | 38 (20/18) | -- | 29 (22/7) | -- |  | |  |
|  | GSH |  | 0.72 ± 0.2 [0.66 – 0.79] | 0.42 – 1.14 | 0.72 ± 0.19 [0.65 – 0.79] | 0-49 – 1.21 | U = 544.0 | | p = 0.929 |
| mOCC | SNR | | 5.9 ± 1 [5 – 6] | 4 - 7 | 6.0 ± 1 [5 – 6] | 4 - 7 | U = 530.0 | | p = 0.769 |
|  | FWHM | | 0.05 ± 0.02 [0.04 - 0.06] | 0.02 – 0.09 | 0.05 ± 0.02 [0.04 – 0.06] | 0.02 – 0.09 | U = 501.5 | | p = 0.529 |
|  | % CRLB GSH | | 12 ± 3 [11 - 13] | 2 - 22 | 12 ± 2 [12 – 13] | 9 - 17 | U = 464.5 | | p = 0.296 |

Abbreviations: DMPFC, dorsomedial prefrontal cortex; mOCC, medial occipital cortex; non-ASD, non-autistic control group; ASD, Autism Spectrum Disorder group; SD, standard deviation; 95% CI, 95% confidence interval; N, number of participants; m, male; f, female; GSH, glutathione; SNR, signal-to-noise ratio; FWHM, full-width-half-maximum; CRLB, Cramer-Rao lower bounds; U, Mann-Whitney test value; p, significance level. ^a^ one-sided t test. Significant differences are in bold (p < 0.05).
