# Supplementary material for: Frontal and occipital brain glutathione levels are unchanged in autistic adults
Source: PLoS One. 2024 Aug 15;19(8):e0308792. doi: 10.1371/journal.pone.0308792 (PMC11326623; doi:10.1371/journal.pone.0308792)
Supplement: S2 Table — (DOCX) [file pone.0308792.s004.docx]

**S2 Table. Voxel tissue proportions for each region and group.**

|  |  |  | non-ASD |  |  | ASD |  | Statistics | | |
| --- | --- | --- | --- | --- | --- | --- | --- | --- | --- | --- |
| Region |  |  | mean ± SD 95% CI | Range |  | mean ± SD 95% CI | Range | |  |  |
|  | N (m/f) |  | 36 (19/17) | -- |  | 26 (20/6) | -- |  | |  |
|  | GM |  | 0.55 ± 0.03 [0.54 – 0.56] | 0.50 – 0.61 |  | 0.54 ± 0.05 [0.52 – 0.56] | 0.45 – 0.62 | U = 425.0 | | p = 0.540 |
| DMPFC | WM |  | 0.22 ± 0.04 [0.21 – 0.24] | 0.16 – 0.31 |  | 0.21 ± 0.04 [0.19 – 0.23] | 0.10 – 0.26 | U = 442.0 | | p = 0.711 |
|  | CSF |  | 0.22 ± 0.05 [0.21 – 0.24] | 0.13 – 0.31 |  | 0.25 ± 0.06 [0.22 – 0.27] | 0.16 – 0.41 | t_(60)_ = -1.634 | | p = 0.108 |
|  | N (m/f) |  | 38 (20/18) | -- |  | 29 (22/7) | -- |  | |  |
|  | GM | | 0.66 ± 0.04 [0.65 – 0.67] | 0.57 – 0.73 |  | 0.65 ± 0.04 [0.23 -0.25] | 0.57 – 0.71 | t_(65)_ = 0.987 | | p = 0.327 |
| mOCC | WM | | 0.24 ± 0.03 [0.23 – 0.25] | 0.19 – 0.32 |  | 0.25 ± 0.03 [0.23 – 0.26] | 0.19 – 0.35 | U = 534.0 | | p = 0.830 |
|  | CSF | | 0.10 ± 0.03 [0.09 – 0.11] | 0.05 – 0.18 |  | 0.10 ± 0.03 [0.09 – 0.12] | 0.04 – 00.16 | U = 486.0 | | p = 0.411 |

Abbreviations: DMPFC, dorsomedial prefrontal cortex; mOCC, medial occipital cortex; non-ASD, neurotypical control group; ASD, Autism Spectrum Disorder group; SD, standard deviation; 95% CI, 95% confidence interval; N, number of participants; m, male; f, female; GM, grey matter; WM, white matter; CSF, cerebrospinal fluid; t, independent samples t test value; U, Mann-Whitney test value; p, significance level.
